# Prominent rash and multisystem inflammatory syndrome in a 29-year-old patient with COVID-19: a case report

**DOI:** 10.1186/s13256-021-03199-1

**Published:** 2021-12-13

**Authors:** Catherine A. Gao, James M. Walter, Jane E. Dematte D’Amico

**Affiliations:** Division of Pulmonary and Critical Care Medicine, Department of Internal Medicine, Northwestern University Feinberg School of Medicine, Arkes Pavilion Suite 1400, 676 North Saint Clair Street, Chicago, IL 60611 USA

**Keywords:** COVID-19, Rash, MIS-C, MIS-A, Case report

## Abstract

**Background:**

Adult patients with coronavirus disease present primarily with respiratory symptoms, but children and some adults may display a more systemic inflammatory syndrome with rash, fever, mucosal changes, and elevated inflammatory biomarkers.

**Case presentation:**

Here, we report the case of a 29-year-old Hispanic patient presenting with significant rash and multisystem inflammation. We describe his clinical course, review dermatological manifestations of coronavirus disease, and summarize the pathophysiology of coronavirus disease-associated multisystem inflammation.

**Conclusion:**

This case should alert physicians to the atypical nature of presenting rash with minimal respiratory symptoms in coronavirus disease.

## Introduction

Adult patients with coronavirus disease 2019 (COVD-19) present primarily with respiratory symptoms such as cough and shortness of breath [1–3]. Children display a more systemic inflammatory syndrome with high fever and Kawasaki’s disease-like findings [4]. Clinical hallmarks include fever, rash, conjunctivitis, distal extremity edema, mucous membrane changes, shock, and high inflammatory markers [5, 6]. This has been called multisystem inflammatory syndrome in children (MIS-C) [7]. The mean age of children diagnosed with MIS-C is 8–9 years [6, 7]. Less common is multisystem inflammatory syndrome in adults (MIS-A) [8]—a syndrome that can include cardiovascular, gastrointestinal, and dermatologic manifestations. Numerous dermatological findings have been described in association with COVID-19 ranging from chilblains, petechiae, purpura, maculopapular rash, and urticaria [9]. Here, we describe a 29-year-old patient with COVID-19 who presented with minimal respiratory symptoms, prominent rash, and marked systemic inflammation.

## Case report

**Fig. 1:**
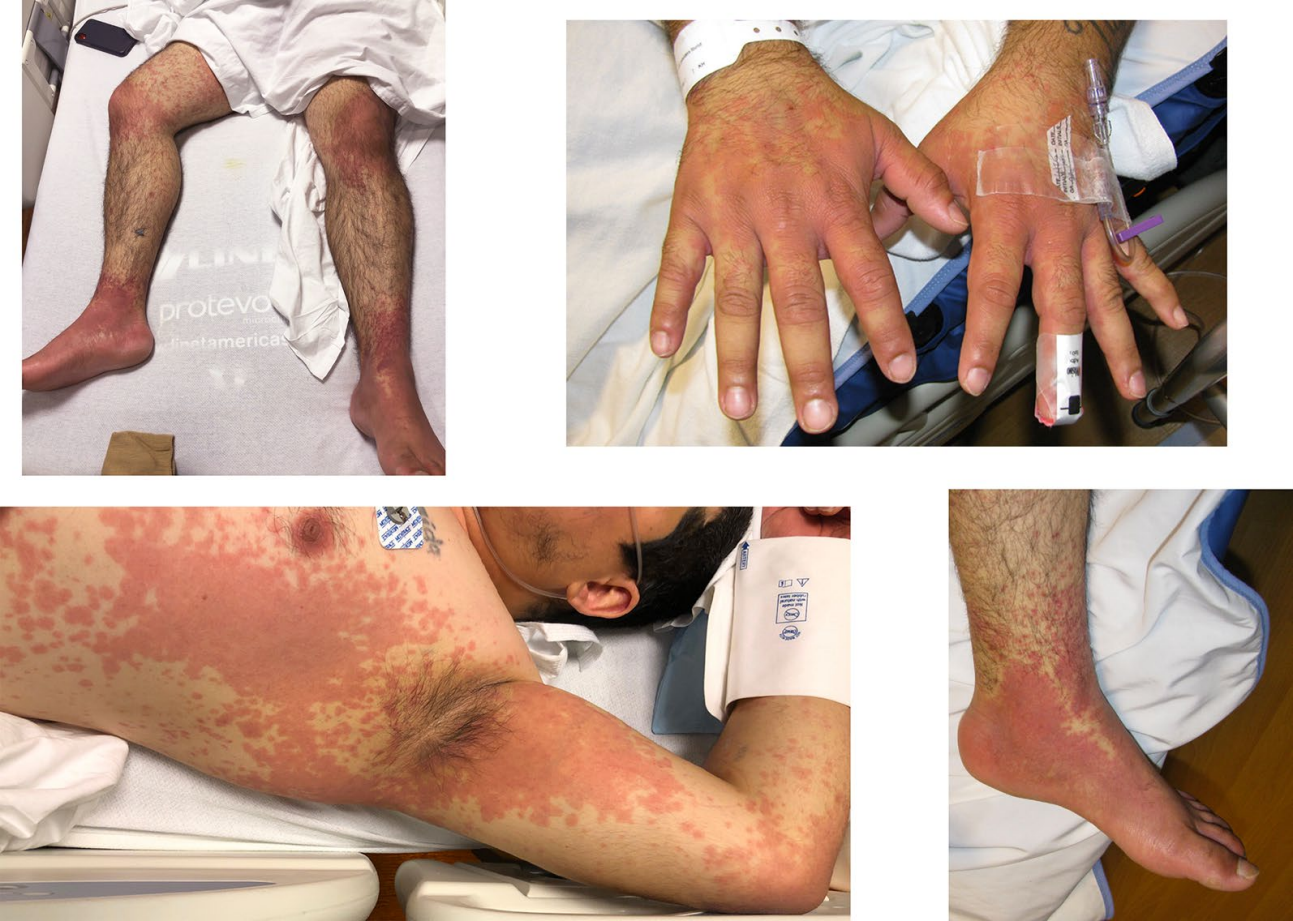
Severe rash on presentation to our hospital, day 6 of symptoms

A 29-year-old Hispanic male was transferred to our tertiary care hospital for worsening rash (Fig. 1), high fevers, and tachycardia in the setting of testing positive for severe acute respiratory syndrome coronavirus 2 (SARS-CoV-2). Vaccines against SARS-CoV-2 were not yet available at the time of his presentation.

He reported first feeling unwell a few days prior to admission, with the gradual onset of fever, palpitations, diarrhea, and abdominal pain. Polymerase chain reaction-based testing for SARS-CoV-2 was positive. He was discharged from the emergency department (ED) after several hours of observation. After discharge, he noticed “a few red dots” on his arms and legs which progressed over the subsequent 12 hours to cover his hands, feet, arms, and legs. The rash was uncomfortable, making it difficult for him to sleep or be touched. Additional symptoms included eye irritation, swelling of the lips, scrotum, hands, and feet, and progressive feedback. He represented to the ED the following day for further evaluation.

The patient had no significant past medical or surgical history. Family history was unremarkable. He was not prescribed any medications and denied smoking, alcohol use, or illicit drugs. He worked full time at a local factory.

Vitals were notable for sinus tachycardia to 170 beats per minute that only mildly improved with a 1 L fluid bolus. Blood pressure was normal. Peripherally measured oxygen saturation was greater than 95% while receiving 2 L/minute supplemental oxygen via nasal cannula.

His exam was notable for a diffuse rash consisting of well-demarcated confluent erythematous patches interspersed with erythematous macules and targetoid lesions (Fig. 1). The rash was most prominent on the chest, abdomen, central and lower back, flank, periaxillary regions, bilateral extensor elbow and knees, scrotum, inguinal folds, hands, and feet in a glove-and-stocking distribution. There was bilateral scleral injection without any crust or exudate. The lips, scrotum, hands, and feet were swollen.

Laboratory evaluation was notable for significantly elevated inflammatory markers [for example, C-reactive protein (CRP) of 385 mg/L; see Table 1], as well as initial eosinophilia, elevated troponin, and mild transaminitis. Computed tomography (CT) of the chest was notable for the absence of focal consolidations or ground-glass opacities commonly seen in patients with COVID-19, although hilar and mediastinal lymphadenopathy was present. A transthoracic echocardiogram was unremarkable.

**Table 1:** Laboratory values near presentation and discharge

Lab name	Lab values near presentation	Lab values near discharge	Normal values
CRP	385.27 ⇧⇧	5.3	0.00–10.00 mg/L
D-dimer	4305 ⇧⇧	401 ⇧	0–229 ng/mL
Ferritin	1514.7 ⇧⇧	975.5 ⇧	24.0–336.0 ng/mL
Procalcitonin	11.33 ⇧⇧	0.95 ⇧	0.00–0.065 ng/mL
Troponin I	0.38 ⇧⇧	0.05	0.00–0.04 ng/mL
LDH	182	191	0–271 Unit/L
WBC	36.5 ⇧	18.1⇧	3.5–10.5 K/UL
HGB	12.4 ⇩	12.9⇩	13.0–17.5 g/dL
PLT	73 ⇩⇩	356	140–390 K/UL
Absolute neutrophils	33.5 ⇧	25.8	1.5–8.0 K/UL
Absolute lymphocytes	0.0 ⇩⇩	1.6	1.0–4.0 K/UL
Absolute monocytes	0.0 ⇩⇩	1.0	0.2–1.0 K/UL
Absolute eosinophils	0.7⇧	0.6	0.0–0.6 K/UL
Sodium	134	134	133–146 mEq/L
Potassium	3.9	3.7	3.5–5.1 mEq/L
Chloride	99	99	98–109 mEq/L
Bicarbonate	24	26	21–31 mEq/L
Calcium	8.8	8.5	8.3–10.5 mg/dL
BUN	16	21	2–25 mg/dL
Creatinine	0.79	0.81	0.60–1.30 mg/dL
Glucose	110	98	65–100 mg/dL
Magnesium	2.2	2.4	1.5–2.7 mg/dL
Phosphorus	3.1	4.2	1.5–2.7 mg/dL
ALT	58⇧	50	0–52 Unit/L
AST	61⇧	26	0–39 Unit/L
Alkaline phos	141⇧	92	34–104 Unit/L
Direct bilirubin	2.7⇧	0.5	34–104 Unit/L
Total bilirubin	3.3⇧	1.1	0.0–1.0 mg/dL
Total protein	5.6⇩	6.2⇩	6.4–8.9 g/dL
Albumin	2.9⇩	2.4⇩	3.5–5.7 g/dL

COVID-19 nasopharyngeal swab—positive

Respiratory pathogen panel—negative

HSV-1, HSV-2 PCR—negative

HH6 quantitative—negative

CMV DNA quant—negative

HIV antigen/antibody—negative

Urine legionella antigen—negative

Urine streptococcus antigen—negative

Parvovirus B19 PCR—negative

EBV PCR—negative

Quantigeron gold—negative

Urine histoplasma antigen—negative

Blood cultures—negative

TTE 6/25/20:

--Study quality: The images were of adequate diagnostic quality and this was a technically difficult study due to patient body habitus.

-The left ventricle is normal in size. There is moderate concentric left ventricular hypertrophy. Left ventricular systolic function is normal. EF = 65% (2D biplane). Left ventricular diastolic function is indeterminate.

-The right ventricle is normal in size. The right atrial pressure is 5 mmHg.

-The right atrium is normal in size.

-There is no mitral valve regurgitation.

-There is no tricuspid valve regurgitation.

-The visualized aorta is normal in size.

The patient was treated with methylprednisone and empiric antibiotics for community-acquired pneumonia (CAP) prior to transfer to our institution. Upon arrival, we started remdesivir (200 mg loading dose followed by 100 mg daily for 9 days) and transitioned his steroids to dexamethasone 6 mg daily (for a total 10-day course). Dermatology was consulted and felt his rash was consistent with a viral exanthem. Other etiologies were considered given the marked acral erythema, but additional infectious tests returned negative (see Table 1). We reviewed potential culprit medications that could cause drug rash and considered drug rash with eosinophilia and systemic symptoms, but felt this diagnosis to be unlikely given the rapid time course and atypical appearance. He was treated with supportive measures including antihistamines and topical steroids. Throughout his course, he noted only mild shortness of breath and never required more than 2 L/minute of supplemental oxygen via nasal cannula to maintain a peripheral oxygen saturation greater than 95%.

**Fig. 2:**
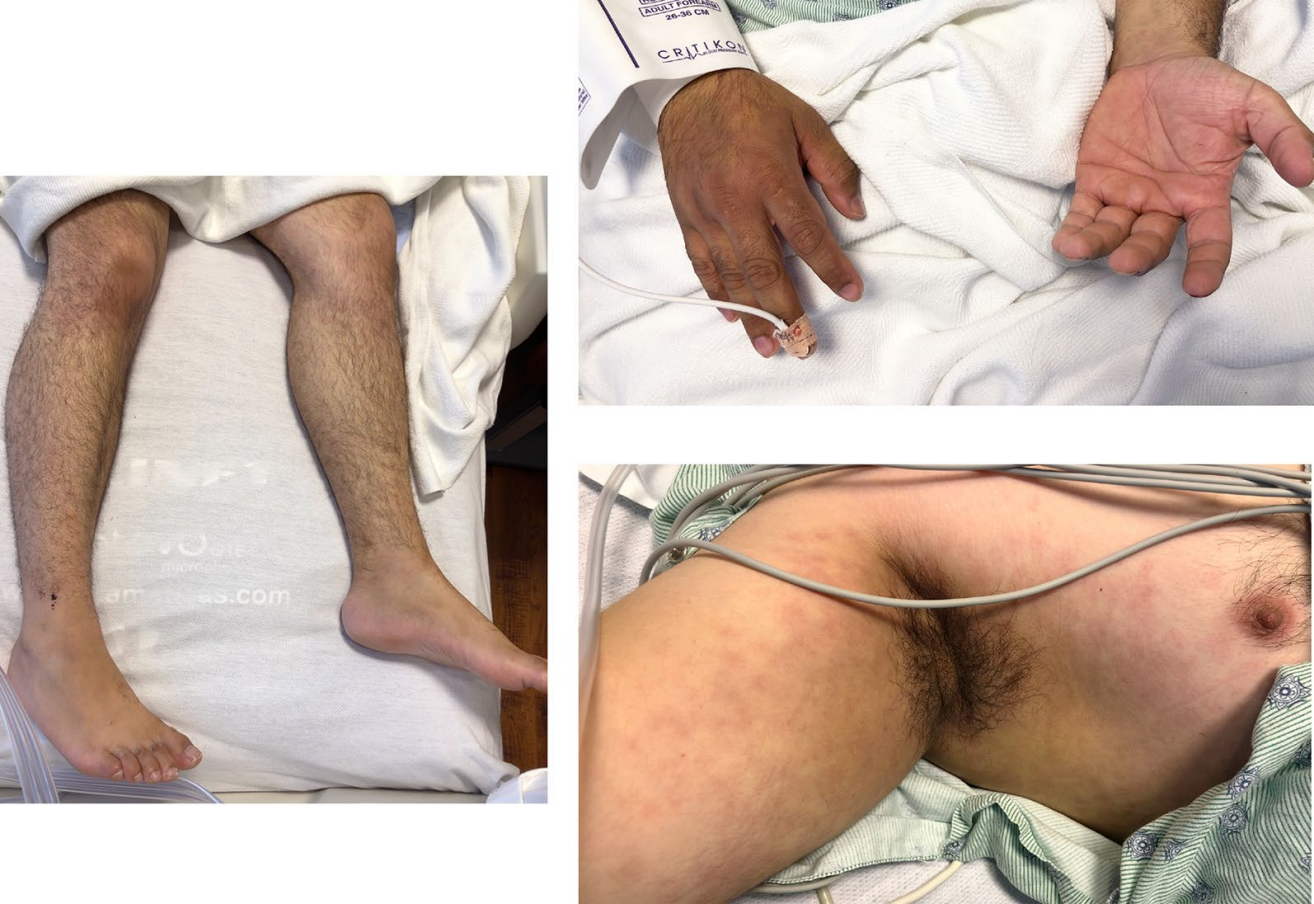
Improvement of rash on day 9 of symptoms

Within a few days, his rash improved significantly (see Fig. 2), as did his diarrhea, vital signs, and inflammatory markers (Table 1). He was discharged home in good health after a 2-week hospital stay.

## Discussion

Adult patients with COVID-19 typically present with respiratory symptoms including cough and shortness of breath. Here, we present an atypical case of COVID-19 in an adult, characterized by a paucity of respiratory symptoms, significant systemic inflammation, and a diffuse rash. Our patient had evidence of systemic inflammation (marked leukocytosis and elevated inflammatory biomarkers) and multi-organ injury: cardiac (elevated troponin), skin (extensive rash), hepatic (mild transaminitis), and pulmonary (mild hypoxemia). The elevation of CRP to several hundred is higher than what is typically described in adults with COVID-19 (10–30 mg/L in the average adult case [10], with an average of 50–100 mg/L described in critically ill patients) [11]. Although he was empirically treated for CAP prior to arrival at our hospital, his mild respiratory symptoms and lack of significant parenchymal abnormalities on chest CT argue against a bacterial coinfection as a driver of his elevated inflammatory biomarkers. Fortunately, our patient improved with supportive measures, in addition to the COVID-19-specific treatments of remdesivir and dexamethasone.

Numerous dermatological findings have been described in patients with COVID-19 [9, 12, 13]. In a recent large review, acral pseudochilblains were the most common finding followed by erythematous maculopapular rashes (although less severe than observed in our patient) [14]. Pathology from a punch biopsy of a patient with chilblains showed a predominantly lymphocytic infiltrate [15]. Clinical registries of COVID-19 are documenting atypical presenting symptoms beyond fever, shortness of breath, and cough. However, the natural history of these extrapulmonary symptoms and their impact on prognosis are unclear. Fortunately, most dermatological manifestations appear to resolve with supportive care [14].

Children are more likely to manifest a multisystem inflammatory syndrome in response to SARS-CoV-2, characterized by fever, markedly elevated inflammatory biomarkers, and extrapulmonary organ systems involvement [4, 7]. Gastrointestinal symptoms, such as the abdominal pain and diarrhea experienced by our patient, are common [16]. Severe systemic inflammation, including various mucocutaneous findings, have been more recently described in adults [17, 18]. How infection with SARS-CoV-2 leads to multisystem inflammation in both children and adults remains unclear. Hypotheses include the stimulation of inflammatory cells such as macrophages, neutrophils, and monocytes, as well as a marked cytokine release causing a hyperimmune, dysregulated host response to infection [19].

## Conclusion

The atypical nature of our patient’s presentation including the paucity of respiratory symptoms should alert clinicians to watch for prominent rash and multisystem inflammation as presenting manifestations of COVID-19 in adults.

## Data Availability

Data sharing not applicable to this article as no datasets were generated or analyzed during the current study.

## Abbreviations

COVID-19: Coronavirus disease 2019
SARS-CoV-2: Severe acute respiratory syndrome coronavirus 2
MIS-C: Multisystem inflammatory syndrome in children
MIS-A: Multisystem inflammatory syndrome in adults
